# Impact Wear of the Protective Cr_3_C_2_-Based HVOF-Sprayed Coatings

**DOI:** 10.3390/ma13092132

**Published:** 2020-05-04

**Authors:** Josef Daniel, Jan Grossman, Šárka Houdková, Martin Bystrianský

**Affiliations:** 1 Institute of Scientific Instruments of the Czech Academy of Sciences, Královopolská 147, 612 64 Brno, Czech Republic; grossman@isibrno.cz; 2 Research and Testing Institute in Plzeň, Tylova 46, 301 00 Plzeň, Czech Republic; houdkova@vzuplzen.cz; 3 Regional Technological Institute, University of West Bohemia, 306 14 Plzeň, Czech Republic; mbyst@rti.zcu.cz

**Keywords:** HVOF, hardmetal, chromium carbide, dynamic impact test, impact wear, thermal spraying

## Abstract

High velocity oxygen-fuel (HVOF) prepared CrC-based hardmetal coatings are generally known for their superior wear, corrosion, and oxidation resistance. These properties make this coating attractive for application in industry. However, under some loading conditions and in aggressive environments, the most commonly used NiCr matrix is not sufficient. The study is focused on the evaluation of dynamic impact wear of the HVOF-sprayed Cr_3_C_2_-25%NiCr and Cr_3_C_2_-50%NiCrMoNb coatings. Both coatings were tested by an impact tester with a wide range of impact loads. The Wohler-like dependence was determined for both coatings’ materials. It was shown that, due to the different microstructure and higher amount of tough matrix, the impact lifetime of the Cr_3_C_2_-50%NiCrMoNb coating was higher than the lifetime of the Cr_3_C_2_-25%NiCr coating. Differences in the behavior of the coatings were the most pronounced at high impact loads.

## 1. Introduction

In many branches of industry, the surfaces of components are exposed to mechanical loading, the influence of an aggressive environment, high temperature, or even a combination of these features. To increase their lifetime, the surface of such components used to be coated by protective coatings. Among others, the technology of thermal spraying earns its position by offering a versatile solution for various kinds of applications and types of loading [1]. Of the required properties, the problem of wear resistance is most often addressed. If combined with high temperatures, the CrC-based hardmetal coatings, deposited by high-velocity oxygen fuel (HVOF) spraying technology, proves its superiority under harsh loading conditions [2,3,4,5]. 

The thermally sprayed chromium-based coatings were evaluated many times, in regards to the used deposition technology [6,7], the influence of deposition parameters [8], and morphology and phase composition of feedstock powder [9]. Their wear resistance under various loading conditions, including the high temperature, was analyzed [3,7,10]. 

Often, the Cr_3_C_2_-25%NiCr coatings are applied to protect the coated parts against erosion, particularly concerning the intended application in the power industry [11,12,13,14,15,16], where erosion by solid particle or water droplet particle is the most curtail for components lifetime. In these works, the mechanism of erosion wear was proposed, taking into account the influence of high temperature and oxidation [13]. It was found that in the aggressive environment, the preferential oxidation of CrC carbides takes place [2,14], leading to a degradation of the coating’s microstructure and increased erosion wear [14]. To increase the resistance against corrosion and oxidation in aggressive environments, the application of CrC-based coatings with alternative matrix compositions was suggested [2,17]. The Cr_3_C_2_-50%NiCrMoNb coating increased corrosion and oxidation resistance due to the optimized matrix composition and comparable abrasive and sliding resistance and wear resistance [2,3].

Despite efforts, the mechanism of erosion wear is still not clearly explained due to the many factors taking part in the wear process. Other than the external conditions, the microstructure, phase composition, and state of internal stress are important. The materials with higher toughness can better accommodate the energy of impacting particles and suffer less brittle cracking [13]. In the case of hardmetals, the toughness of the material can be optimized by an increase of the soft metal matrix [18]. 

As surface dynamic loading appeared in various kinds of components, the need for its evaluation has led to the development of the unambiguous test. Knotek et al. developed a method of the dynamic impact test [19], originally focused mainly on the analysis of thin-film systems. During the dynamic impact test, the surface of the specimen is repeatedly impacted by ball indenter. Load force is well-defined and impact frequency is used to be a constant value. Using a dynamic impact test, it is possible to determine the impact wear or impact lifetime of the tested specimen [20,21,22,23,24,25].

The dynamic impact test was consequently adopted by the researchers focusing on the evaluation of thick thermally sprayed coatings [26,27,28], although the number of studies is rather limited. David et al. compared the HVOF sprayed coatings based on the numbers of impacts at various loads end evaluated the fracture modes [27]. Bobzin et al. analyzed the Cr_3_C_2_-NiCr coating impact wear by evaluation of impact craters created by ~ 10^5^–10^6^ impacts to obtain the coating failure modes and mechanisms [26]. In a recent study, Kiilakoski et al. evaluated the fatigue life of ceramic coatings exploiting low-energy impact conditions [28]. In this study, the impact wear was successfully related to coating cavitation wear resistance. 

Although the first attempts to evaluate the impact wear resistance of several thermally sprayed coatings were completed, complex study of the impact wear of the Cr_3_C_2_-25%NiCr HVOF-sprayed coating including coating impact lifetime under various impact load remains absent. Additionally, the methodology of results evaluation needs to be established. The aim of this work is the complex study of impact wear and dynamic impact load limits of the HVOF-sprayed Cr_3_C_2_-25%NiCr and Cr_3_C_2_-50%NiCrMoNb coatings. Results obtained by impact testing were compared and discussed with respect to the microstructure of the coatings.

## 2. Experimental 

### 2.1. Sample Preparation

The HVOF-sprayed coatings were deposited onto the flat surface samples of high-speed steel 5 mm thick cylindrical samples with a diameter of 20 mm. The coated surface was grit blasted before spraying to ensure sufficient adhesion of the coating to the substrate surface, using Al_2_O_3_; F20 abrasive media. Commercially available powders were used to spray the coatings—Amperit 588.074 was used for the preparation of the Cr_3_C_2_-25%NiCr coating and Amperit 595.074 was used for the preparation of the Cr_3_C_2_-50%NiCrMoNb coating. Both coatings were deposited using the HP/HVOF TAFA JP5000 spraying device (Praxair Surface Technologies, Indianapolis, IN, USA). The thicknesses of the sprayed coatings were set to 400 µm. The spraying parameters are summarized in Table 1. 

### 2.2. Dynamic Impact Test

**Figure 1:**
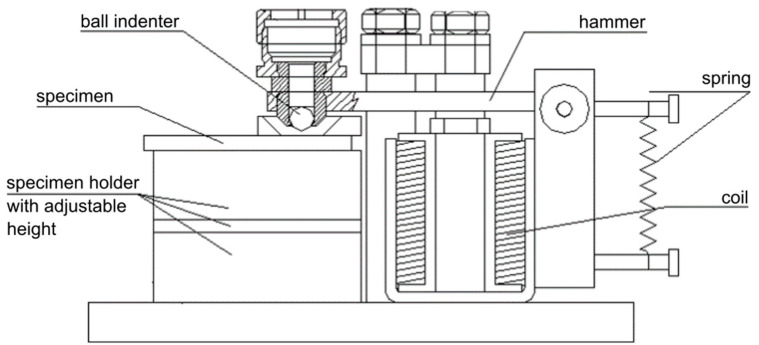
Dynamic impact tester developed at ISI CAS.

Coatings behaviour under dynamic impact load was investigated using impact tester developed at the Institute of Scientific Instrumentation CAS in Brno, Czech Republic [20,21]. The tester is schematically described in Figure 1. 

The impact hammer was driven electromagnetically; the amplitude of the loading force was regulated by an electric current in the solenoids. The ball-shaped tungsten carbide with a diameter of 5 mm was used as an impact indenter. The impacting frequency was set to 8 Hz. The indenter ball was adjusted to the unworn contact side before every test. Impact testing was carried out with the impact loads of 150 N, 200 N, 400 N and 600 N. Speed of the ball indenter before contact with specimen was in the range of 0.4 m·s^−1^ for the impact load of 150 N to 0.9 m·s^−1^ for the impact load of 600 N. Number of impacts were in the range from 1 up to 250,000. Every test was repeated three times for the elimination of surface inhomogeneity. All of the impact tests were carried out at room temperature.

The depth and radius of the impact craters were measured by the profilometer Talystep (Taylor Hobson, UK). The size and surface morphology of the impact craters were determined using confocal microscope Lext OLS 3100 (Olympus, Japan). Based on the work of Engel et al. for the physical vapor deposited (PVD) coatings [22], the methodology of critical crater volume determination was adapted and applied for evaluation of impact wear of HVOF sprayed coatings. In detail, the methodology is described in the next chapter.

## 3. Results and Discussion

### 3.1. CrC-Based Coatings Structure and Properties

**Figure 2:**
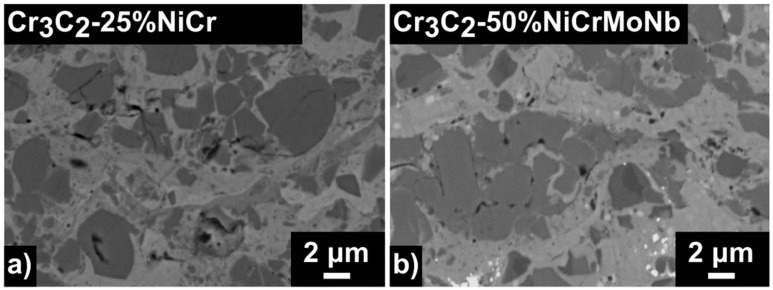
The microstructure of the studied coatings. Comparison of (**a**) the Cr_3_C_2_-25%NiCr coating and (**b**)the Cr_3_C_2_-50%NiCrMoNb coating.

Structure, phase composition, and mechanical and tribological properties of both of the studied HVOF-sprayed Cr_3_C_2_-25%NiCr and Cr_3_C_2_-50%NiCrMoNb coatings were described in detail by Houdkova et al. [2]. The Cr_3_C_2_-25%NiCr coating contained 25% of the Ni-Cr-based matrix, which surrounded carbide Cr_3_C_2_, Cr_7_C_3_, and (Cr, Ni)_7_C grains (Figure 2a). 

Moreover, some amount of carbon was also incorporated into the matrix as a result of carbon dissolution during the spraying process. This various carbon amount in the matrix is represented as the shades of grey in the matrix in Figure 2a. On the other hand, the Cr_3_C_2_-50%NiCrMoNb coating contained 50% of the Ni–Cr–Mo–Nb-based matrix (Figure 2b). A matrix with a higher amount of dissolved carbon surrounded Cr_3_C_2_ and (Mo, Ni, Cr)_7_C_3_ grains and Nb–C precipitates. Furthermore, the Ni-based matrix also contained fcc precipitates with small crystallites and a bigger lattice parameter, the so-called γ-phase [2].

In [1], the mechanical properties and wear resistance of both coatings were compared. It was shown that despite the slightly lower hardness of Cr_3_C_2_-50%NiCrMoNb, caused by the higher amount of metallic matrix, its sliding wear resistance and coefficient of friction were comparable to the Cr_3_C_2_-25%NiCr coating.

### 3.2. Impact Behaviour of the CrC-Based Coatings

**Figure 3:**
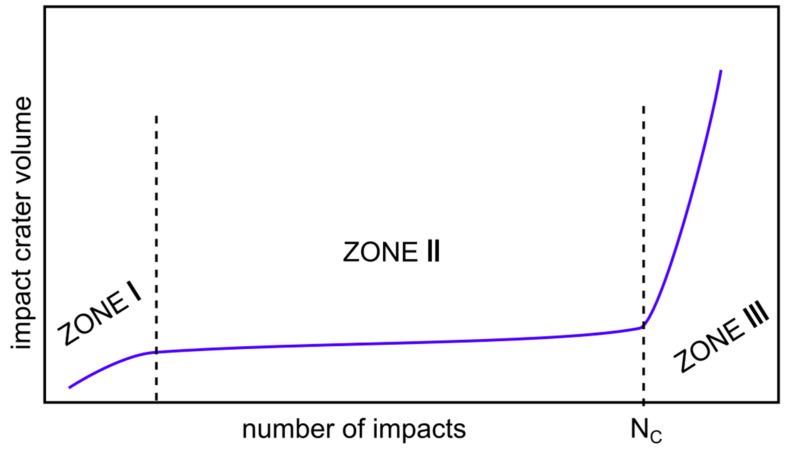
The scheme of the loading curve divided into three zones. Adopted from [22] and modified.

The basic result of impact testing is the loading curve-dependence of the impact crater volume on the number of impacts [20, 21, 22]. According to Engel et al., the loading curve can be divided into three zones, as is schematically drawn in Figure 3 [22]. 

At the beginning of Zone I, the first impact causes deformation of the specimen. Any subsequent impact causes further deformation and the impact crater volume increases. However, the increment of the impact crater volume after the second impact is lower than increment after the first impact. As the number of impacts increases further, the increment of the impact crater volume decreases, and the system transits from Zone I to Zone II. This transition occurs at the low number of impacts and, for the investigation of the coating, impact lifetime is not important. In Zone II, the energy from the indentor is dissipated mainly in the form of inner stress in the specimen and just a minimal increase in crater volume is observed. In some materials, the formation of pile-ups around the impact crater can be observed. These pile-ups can be formed due to the material transport induced by impacting or due to the substrate deformation [29,30]. As the number of impacts increases further, the amount of stress increases up to a certain critical value. The number of impacts corresponding to this critical value, which is the critical number of impacts N_C_, corresponds to the impact load limit. The critical number of impacts forms a boundary between Zone II and Zone III. 

In Zone III, the impact crater volume rapidly increases with an increasing number of impacts, and coating tends to fail. The thin PVD coatings exhibit damage, delamination, and revealing of the substrate on the impact crater bottom and their N_C_ might be evaluated using both the rapid increase of the carter volume and the using the analyses of the coating delamination [11]. However, in the case of the thick HVOF coatings, no delamination or revealing of the substrate was observed. Thus, in this work, N_C_ was evaluated only using the rapid increase of the impact crater volume.

**Figure 4:**
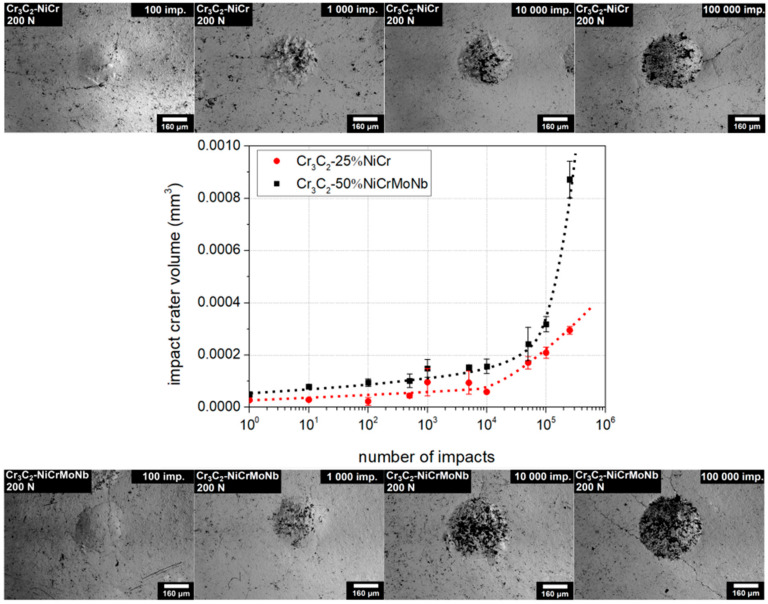
Comparison of the loading curves and impact craters of the studied coatings. The case for impact load of 200 N. Dotted lines were added as a visual guide.

The impact wear was evaluated and compared for both CrC-based coatings at 150 N, 200 N, 400 N, and 600 N and analyzed. In Figure 4, the evolution of impact crater volumes (in mm^3^) on the number of impacts are shown for the impact load 200 N. 

Observed volumes of the impact craters of the Cr_3_C_2_-50%NiCrMoNb coating (black marks) were slightly higher than the volume of the Cr_3_C_2_-25%NiCr coating (red marks). Transition to the Zone III (rapid increase of the impact crater volume) occurred in the case of Cr_3_C_2_-25%NiCr coating earlier than in the case of the Cr_3_C_2_-50%NiCrMoNb coating. Thus, the critical number of impacts of the Cr_3_C_2_-25%NiCr coating was, for impact load 200 N, lower than the critical number of impacts of the Cr_3_C_2_-50%NiCrMoNb coating. Impact craters of the Cr_3_C_2_-25%NiCr and Cr_3_C_2_-50%NiCrMoNb coatings related to the 100, 1000, 10,000, and 100,000 impacts and an impact load of 200 N are also depicted in Figure 4. One can see that the dimension of the Cr_3_C_2_-50%NiCrMoNb impact craters was slightly higher than the dimension of Cr_3_C_2_-25%NiCr craters.

**Figure 5:**
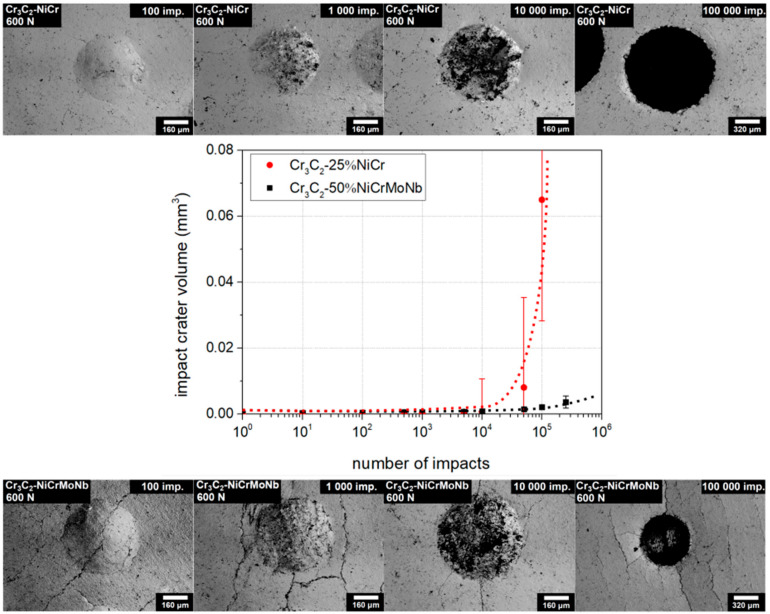
Comparison of the loading curves and impact craters of the studied coatings. The case for impact load of 600 N. Dotted lines were added as a visual guide.

The loading curves of the Cr_3_C_2_-25%NiCr and Cr_3_C_2_-50%NiCrMoNb coatings obtained with a load of 600 N are depicted in Figure 5. Volumes of the impact craters of both coatings in Zones I and II were comparable. The rapid increase of the impact crater volume of the Cr_3_C_2_-25%NiCr coating (red marks) was observed at a lower number of impacts than for the Cr_3_C_2_-50%NiCrMoNb coating (black marks). Moreover, the impact craters of the Cr_3_C_2_-25%NiCr coating created by ≳ 10,000 impacts exhibited a large dispersion of the volume. Impact craters of the Cr_3_C_2_-25%NiCr and Cr_3_C_2_-50%NiCrMoNb coatings related to the 100, 1000, 10,000, and 100,000 impacts and a load of 600 N are also depicted in Figure 5.

Craters related to 100,000 impacts are depicted with twice the magnification of the others. The impact crater of the Cr_3_C_2_-25%NiC coating related to the 100,000 impacts was much larger than the corresponding impact crater of the Cr_3_C_2_-50%NiCrMoNb coating. Parallel microcracks through all micrographs in the case of the Cr_3_C_2_-50%NiCrMoNb coating are artefacts of mechanical damage of the coating.

**Figure 6:**
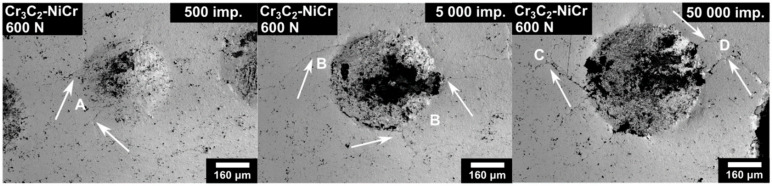
Dynamics of the microcracks spreading illustrated on the Cr_3_C_2_-25%NiC coating.

Both the tested coatings exhibited radial cracking on the edge of the impact crater. The microcracks spread and expanded as the number of impacts increased. The dynamics of microcrack spreading can be explained using Figure 6. 

The impact craters of the Cr_3_C_2_-25%NiC coating related to 500, 5000, and 50,000 impacts are shown. At first, small radial microcracks were created on the edge of the impact crater (A). As the number of impacts increased, the number of radial microcracks grew and the size of the microcracks achieved the order of 100 µm (B). With further increase of impacts and thereby the amount of energy from the tester, the microcracks stopped to spread in the radial direction and tended to ramify (C). Some microcracks connected and created a closed area (D). 

The first cracking, in the case of the dynamic load of 200 N, was observed in both coatings after 500 impacts. Both coatings exhibited similar microcrack size and density at the high number of impacts. However, in the case of the impact load of 600 N, the dynamics of microcrack spreading were different. The Cr_3_C_2_-25%NiCr coating exhibited the first indication of cracking after 10 impacts and after 50,000 impacts (see Figure 6) the 100–200 µm length microcracks ramified and connected themselves. On the other hand, the Cr_3_C_2_-50%NiCrMoNb coating exhibited the first indication of cracking after 500 impacts, and more than 300 µm length microcracks tended to create a connection at 250,000 impacts.

The process of evaluation of the critical number of impacts had to be slightly modified for the thick HVOF-sprayed coatings. The critical number of impacts, in the case of the thin PVD coatings, is estimated as the highest number of impacts before coating failure, like delamination or revealing of the substrate [19,20,21,22,30,31]. Such a critical number of impacts corresponds to the beginning of Zone III in the loading curve. In the case of HVOF-sprayed coatings, the critical number of impacts was estimated also as a beginning of Zone III in the loading curve. However, in the case of the observed large dispersion of the value of impact crater volume as shown in Figure 5, the critical number of impacts was estimated as the highest number of impacts before this large dispersion occurred. A similar methodology was used to evaluate the critical crater volumes for both coatings, tested at all of the impact loads.

**Figure 7:**
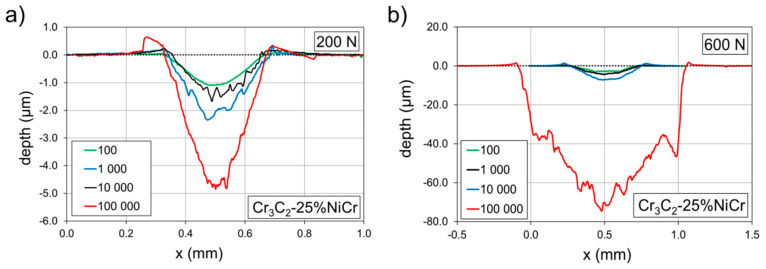
Profiles of the impact craters of the Cr_3_C_2_-25%NiCr coating. Comparison of the impact loads of (**a**) 200 N and (**b**) 600 N.

Profiles of the impact craters of the Cr_3_C_2_-25%NiCr coating formed by the 100, 1000, 10,000, and 100,000 impacts are compared in Figure 7. 

All profiles are related to craters depicted in Figure 4 and Figure 5. Impact craters created by an impact load of 200 N (Figure 7a) and 100,000 impacts exhibited only small pile-ups on the edge of the impact craters. Since the depth of the impact crater was ~ 1% of the coating thickness, observed pile-ups were induced by the material transport rather than by the substrate deformation. Similarly, only small pile-ups were detected in the case of impact craters created by the load of 600 N (Figure 7b). The rapid increase of the impact crater after 100,000 impacts in Figure 7b corresponds to the rapid increase of the crater volume in Figure 5. The profile of this impact crater was considerably rougher in comparison to the other profiles.

**Figure 8:**
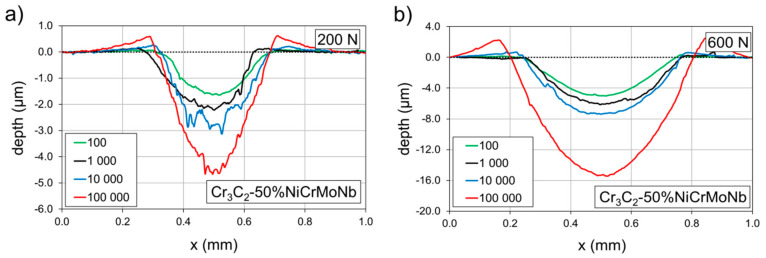
Profiles of the impact craters of the Cr_3_C_2_-50%NiCrMoNb coating. Comparison of the impact loads of (**a**) 200 N and (**b**) 600 N.

Profiles of the impact craters of the Cr_3_C_2_-50%NiCrMoNb coating created by the 100, 1000, 10,000, and 100,000 impacts are shown in Figure 8. 

As in the previous case, all profiles are related to craters depicted in Figure 4 and Figure 5. Craters formed under an impact load of 200 N (Figure 8a) and 10,000 and 100,000 impacts exhibited huge pile-ups on their edges. Similarly, craters prepared using an impact load of 600 N (Figure 8b) and by 10,000 and 100,000 impacts exhibited considerable pile-ups. Profile of the impact crater of the Cr_3_C_2_-50%NiCrMoNb coating prepared with a load of 600 N and 100,000 impacts was smooth in comparison with the corresponding impact crater of the Cr_3_C_2_-25%NiCr coating.

It was shown that the bonding of carbide Cr_3_C_2_ grains to the surrounding NiCr matrix in the Cr_3_C_2_-25%NiCr coating is a critical point with respect to the coating’s wear resistance [4,5]. The formation of microcracks along the carbide grain boundaries and pulling-out of individual grains during wear tests were observed [2,7], leading to loss of coatings material. Therefore, we assume that the observed coarse profile of the crater prepared by the impact load of 600 N and 100,000 impacts (Figure 7b) in the Cr_3_C_2_-25%NiCr coating also resulted from the pulling-out of carbide grains. This can be explained as follows: when the number of impacts (or energy supplied from tester into the tested system) is sufficiently high, the stress in the material tends to form microcracks. Microcracks spread along the grain boundaries and cause the pulling-out of grains. Moreover, due to the random structure, microcracks also spread in random directions and pull-out a random amount of material. Thus, the volume of the crater was not the same for the same number of impacts and the average value of the volume of such impact craters exhibited large dispersion. This was observed in the case of the Cr_3_C_2_-25%NiCr coating and the load of 600 N (see Figure 5). 

This claim is in good agreement with the dynamic of microcrack spreading observed at the surface of the coating and described above. The surface microcracks on the Cr_3_C_2_-25%NiCr coating prepared by the impact load of 600 N tended to connect at 50,000 impacts and create a closed area which can be easily pull-out. This microcrack spreading and thereby amount of this closed area is random and thus, the average value of the volume of such impact craters, exhibits a large dispersion. 

**Figure 9:**
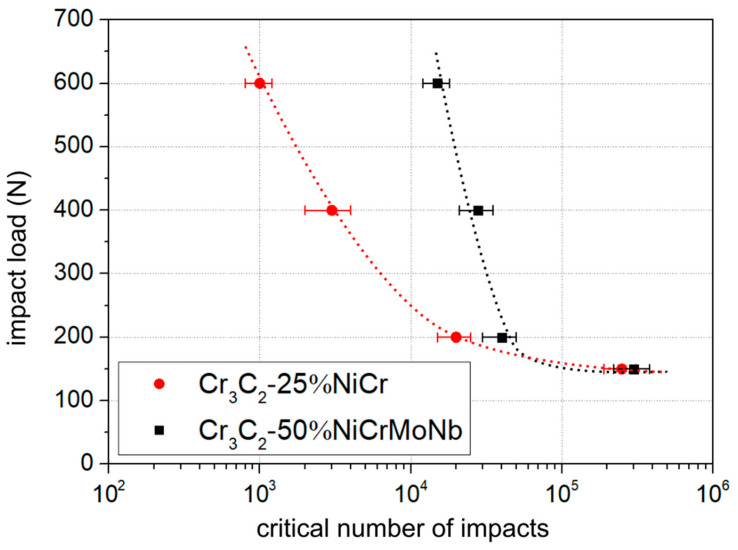
Dependence of the critical number of impacts on the used impact load. Comparison of the Cr_3_C_2_-25%NiCr and Cr_3_C_2_-50%NiCrMoNb coatings. Dotted lines were added as a visual guide.

A critical number of impacts of both coatings was determined for all the impact loads used—150 N, 200 N, 400 N, and 600 N. Dependence of the N_C_ on the impact load, the Wöhler-like curve, is depicted in Figure 9. 

The Cr_3_C_2_-25%NiCr coating (red marks) exhibited a lower impact load limit than the coating Cr_3_C_2_-50%NiCrMoNb (black marks). The difference in the impact load limit was the most distinct in the impact load of 600 N. The difference in the impact load limits became smaller as the impact load decreased. Finally, for the impact load of 150 N, the impact lifetime of both coatings was comparable.

A possible explanation of the different impact lifetimes is based on the different microstructure of the tested coatings. The coating Cr_3_C_2_-50%NiCrMoNb exhibited a higher number of soft matrices. Thus, energy supplied from tester to the coating was dissipated at first in the form of deformation and transport of material (pile-ups observed in Figure 8). The formation of microcracks required more energy, i.e., a higher number of impacts. On the other hand, the Cr_3_C_2_-25%NiCr coating exhibited a lower number of matrices and the possibility of material transport was limited. Supplied energy was rather dissipated into the formation of microcracks and the pulling-out of material. This resulted in a rapid increase in the impact crater volume at the lower number of impacts than in the case of the Cr_3_C_2_-50%NiCrMoNb coating. Thus, the Cr_3_C_2_-50%NiCrMoNb coating exhibited a higher critical number of impacts than the Cr_3_C_2_-25%NiCr coating.

The amount of energy supplied from tester to the tested system decreased with decreasing impact load. In the case of the lowest impact load of 150 N, the amount of supplied energy was insufficient to the formation of microcracks or even to the induction of material transport. Therefore, no differences in the impact load limit of both coatings were observed in case of the lowest impact load.

## 4. Conclusions

Impact wear of two HVOF-sprayed coatings—the Cr_3_C_2_-25%NiCr and the Cr_3_C_2_-50%NiCrMoNb—were investigated. The impact loads of 150 N, 200 N, 400 N, and 600 N were used. The results can be summarized as follows:

- The critical number of impacts of the thick HVOF-sprayed coatings was estimated using the loading curve and dispersion of values of the average volume of the impact crater.
- Observed dispersion of values of the average volume of the impact crater was the consequence of the different spread of microcracks in the coatings.
- The Cr_3_C_2_-50%NiCrMoNb coating exhibited, under the impact load of 200 N, a higher volume of impact craters, and the impact lifetime was nevertheless higher than for the Cr_3_C_2_-25%NiCr coating.
- The Cr_3_C_2_-50%NiCrMoNb coating exhibited higher impact lifetime than the Cr_3_C_2_-25%NiCr coating, probably due to the higher number of ductile metallic matrices.
- The difference between the impact lifetimes of the coatings was the most pronounced at high impact loads.

## Figures and Tables

**Table 1:** Coatings deposition parameters.

Material	Cr_3_C_2_-25%NiCr	Cr_3_C_2_-50%NiCrMoNb
Feedstock	Amperit 588.074	Amperit 595.074
Oxygen	823 L/min	872 L/min
Fuel	25.7 L/h	21.7 L/h
Barrel length	100 mm	150 cm
Spray distance	360 mm	330 mm
Traverse speed	250 mm/s	250 mm/s
Feed rate	70 g/min	76 g/min
Carrier gas	Nitrogen, 6 L/min	Nitrogen, 6 L/min
Offset	6 mm	6 mm
